# Supplementary material for: Peptidoglycan Endopeptidase from Novel Adaiavirus Bacteriophage Lyses Pseudomonas aeruginosa Strains as Well as Arthrobacter globiformis and A. pascens Bacteria
Source: Microorganisms. 2023 Jul 26;11(8):1888. doi: 10.3390/microorganisms11081888 (PMC10458142; doi:10.3390/microorganisms11081888)
Supplement: Supplementary file 1 [file microorganisms-11-01888-s001.zip › microorganisms-2515141-supplementary.pdf]

Supplementary material

Supplementary Table S1. Annotation of Pseudomonas virus Hadban genes.

ORF	start	end	The best similar protein	E-value	Aa similarity with Adaia
**1**	87	428	hypothetical protein PBI_ATRAXA_1 [Arthrobacter phage Atraxa]	4.9e-50	93.9
**2**	421	654	hypothetical protein PBI_ADAIA_2 [Arthrobacter phage Adaia]	2.70E-54	97.4
**3**	651	2057	COG4626 phage terminase, large subunit [uncultured Caudovirales phage]	3.10E-168	99.2
**4**	2069	3190	portal protein [Siphoviridae sp.]	2.90E-122	98.1
**5**	3183	4796	major capsid and protease fusion protein [Gordonia phage Schiebs]	1.60E-152	98.3
**6**	4861	5205	head-to-tail adaptor [Gordonia phage Coeur]	4.4E-39	93.6
**7**	5205	5588	hypothetical protein PBI_ADAIA_7 [Arthrobacter phage Adaia]	1.10E-73	99.2
**8**	5592	6017	tail tube protein family protein [Bacteriophage sp.]	6.60E-49	95.1
**9**	6020	6409	putative tail component	2.00E-51	100
**10**	6423	6716	tail assembly chaperone [Arthrobacter phage Adaia]	1.80E-34	100
**11**	6734	6856	tail assembly chaperone [Arthrobacter phage Adaia]	1.70E-31	100
**12**	6857	8773	tape measure protein [Arthrobacter phage Adaia]	4.60E-214	99.8
**13**	8773	10035	minor tail protein [Arthrobacter phage Atraxa]	8.70E-162	99.0
**14**	10035	11648	minor tail protein [Arthrobacter phage Adaia]	7.30E-196	76.6
**15**	11645	11791	hypothetical protein SEA_PRAIRIE_33 [Arthrobacter phage Prairie]	4.20E-21	-
**16**	11775	12440	lysin A [Arthrobacter phage Adaia]	3.40E-60	96.4
**17**	12451	12678	hypothetical protein PBI_ATRAXA_16 [Arthrobacter phage Atraxa]	2.80E-32	100
**18**	12668	12970	hypothetical protein PBI_ATRAXA_17 [Arthrobacter phage Atraxa]	9.10E-57	99
**19**	13015	13200	hypothetical protein PBI_ADAIA_18 [Arthrobacter phage Adaia]	9.00E-44	98.4
**20**	13235	13468	hypothetical protein PBI_ADAIA_19 [Arthrobacter phage Adaia]	6.10E-51	76.9
**21**	13465	13683	hypothetical protein PBI_ADAIA_20 [Arthrobacter phage Adaia]	8.70E-49	100
**22**	13680	13865	hypothetical protein PBI_ADAIA_21 [Arthrobacter phage Adaia]	4.60E-42	98.4
**23**	13862	14365	nothing, but aa 1-61 identical with Arthrobacter JUb119 MCS3494086		
**24**	14426	14632	nothing, but 27/68 aa identical with hypothetical protein of Arthrobacter		
**25**	14635	14823	hypothetical protein PBI_ADAIA_24 [Arthrobacter phage Adaia]	4.50E-44	88.9
**26**	14813	15031	hypothetical protein PBI_ADAIA_25 [Arthrobacter phage Adaia]	1.20E-30	48.5
**27**	15859	16149	hypothetical protein PBI_ADAIA_27 [Arthrobacter phage Adaia]	5.20E-55	80.0
**28**	16184	16429	HNH endonuclease [Siphoviridae sp.]	2.10E-28	98.8
